# Impact of Air Pollution on the Ocular Surface and Tear Cytokine Levels: A Multicenter Prospective Cohort Study

**DOI:** 10.3389/fmed.2022.909330

**Published:** 2022-07-06

**Authors:** Ran Hao, Mingzhou Zhang, Liming Zhao, Yang Liu, Min Sun, Jing Dong, Yanhui Xu, Feng Wu, Jinwen Wei, Xiangyang Xin, Zhongping Luo, Shuxuan Lv, Xuemin Li

**Affiliations:** ^1^Department of Ophthalmology, Peking University Third Hospital, Beijing, China; ^2^Beijing Key Laboratory of Restoration of Damaged Ocular Nerve, Peking University Third Hospital, Beijing, China; ^3^Department of Ophthalmology, Beijing Fengtai Hospital, Beijing, China; ^4^Department of Ophthalmology, Daqing Oilfield General Hospital, Daqing, China; ^5^Department of Ophthalmology, Huabei Petroleum General Hospital, Cangzhou, China; ^6^Department of Ophthalmology, The First Affiliated Hospital of Baotou Medical College, Inner Mongolia, China; ^7^Department of Ophthalmology, Hebei Provincial Eye Hospital, Shijiazhuang, China; ^8^Department of Ophthalmology, Fuyang Hospital Affiliated to Anhui Medical University, Fuyang, China; ^9^Department of Ophthalmology, Inner Mongolia Autonomous Region Xilingol League Hospital, Inner Mongolia, China; ^10^Department of Ophthalmology, Inner Mongolia Baogang Hospital, Inner Mongolia, China; ^11^Department of Ophthalmology, Tongliao City Ke’erqin Zuoyi Zhongqi People’s Hospital, Inner Mongolia, China; ^12^Department of Ophthalmology, Yongqing People’s Hospital, Langfang, China

**Keywords:** air pollution, dry eye disease, meibomian gland, ocular surface, tear cytokine

## Abstract

**Purpose:**

To assess air pollution-induced changes on ocular surface and tear cytokine levels.

**Methods:**

As a prospective multicenter cohort study, 387 dry eye disease (DED) participants were recruited from five provinces in China and underwent measurements of ocular surface disease index (OSDI), Schirmer’s I test (ST), tear meniscus height (TMH), tear film break-up time (TBUT), corneal fluorescein staining (CFS), meibomian gland (MG) function, and tear cytokines. The associations between ocular surface parameters and exposure to particulate matter (PM), ozone (O_3_), nitrogen dioxide (NO_2_), and sulfur dioxide (SO_2_) for 1 day, 1 week, and 1 month before the examination were analyzed in single- and multi-pollutant models adjusted for confounding factors.

**Results:**

In the multi-pollutant model, the OSDI score was positively correlated with PM with diameter ≤2.5 μm (PM_2.5_), O_3_, and SO_2_ exposure [PM_2.5_: β (1 week/month) = 0.229 (95% confidence interval (CI): 0.035–0.424)/0.211 (95% CI: 0.160–0.583); O_3_: β (1 day/week/month) = 0.403 (95% CI: 0.229–0.523)/0.471 (95% CI: 0.252–0.693)/0.468 (95% CI: 0.215–0.732); SO_2_: β (1 day/week) = 0.437 (95% CI: 0.193–0.680)/0.470 (95% CI: 0.040–0.901)]. Tear secretion was negatively correlated with O_3_ and NO_2_ exposures but positively correlated with PM_2.5_ levels. Air pollutants were negatively correlated with TBUT and positively related with CFS score. Besides SO_2_, all other pollutants were associated with aggravated MG dysfunction (MG expression, secretion, and loss) and tear cytokines increasement, such as PM_2.5_ and interleukin-8 (IL-8) [β (1 day) = 0.016 (95% CI: 0.003–0.029)], PM with diameter ≤10 μm (PM_10_) and IL-6 [β (1 day) = 0.019 (95% CI: 0.006–0.033)], NO_2_ and IL-6 [β (1 month) = 0.045 (95% CI: 0.018–0.072)], among others. The effects of air pollutants on DED symptoms/signs, MG functions and tear cytokines peaked within 1 week, 1 month, and 1 day, respectively.

**Conclusion:**

Increased PM_2.5_, O_3_, and SO_2_ exposures caused ocular discomfort and damage with tear film instability. PM_10_ exposure led to tear film instability and ocular injury. PM, O_3_, and NO_2_ exposures aggravated MG dysfunction and upregulated tear cytokine levels. Therefore, each air pollutant may influence DED *via* different mechanisms within different time windows.

## Introduction

The increasing levels of environmental pollution worldwide pose a serious threat to public health (1–5). Air pollution can cause an extensive range of respiratory and cardiovascular diseases (3–9), metabolic diseases (10), strokes (11), sudden infant death syndrome (12), and even an increasement of mortality (13). According to World Health Organization (WHO), particulate matter (PM), ozone (O_3_), nitrogen dioxide (NO_2_), and sulfur dioxide (SO_2_) are the most significant pollutants.

The ocular surface is constantly and directly exposed to the external environment; however, the previous researches assessed dry eye disease (DED) only through binary symptoms or diagnosis (2, 14, 15). The importance of inflammation and tear cytokines on the pathogenesis of DED has been highlighted by the Tear Film and Ocular Surface Society International Dry Eye Workshop II (TFOS DEW II) (16). In addition, exposure to high levels of air pollutants were reported to cause ocular surface inflammation and tear cytokines increasement in animal models (17, 18). However, clinical validation about the fluctuations in tear cytokine levels exposure to air pollutants has not been reported until now.

In this study, we evaluated the different effects of various air pollutants, PM with diameter ≤2.5 μm (PM_2.5_) and diameter ≤10 μm (PM_10_), O_3_, NO_2_, and SO_2_, on the clinical characteristics and tear cytokines of DED. We aim to identify which air pollutant mainly influence ocular surface and the time window from exposure to air pollution to DED occurrence.

## Materials and Methods

### Study Participants and Design

In this multicenter prospective cohort study, individuals were recruited from 11 hospitals across five provinces in China, namely, Beijing, Hebei, Heilongjiang, Anhui, and Inner Mongolia, from 1 February 2019, through 31 January 2020. Participants aged 20–80 years were eligible for enrollment. DED was defined according to the TFOS DEW II standards: ocular surface disease index (OSDI) ≥13 and tear film break-up time (TBUT) <10 s, or ocular surface staining (>5 corneal spots and >9 conjunctival spots) (19). Subjects with another ocular surface abnormality, with a history of contact lens use or refractive surgery, with glaucoma medications usage, underwent ocular surgery within the past 6 months were excluded from the study. Participants in each hospital were examined by the same trained doctor, including the ocular surface health assessments and tear cytokine level measurements. The DED patients were stratified by severity grading scheme (level 1–4) according to International Dry Eye WorkShop (2007) (20). Informed consents were obtained from all participants. The study adhered to the Declaration of Helsinki and was approved by the Peking University Third Hospital Ethics Committee (No. M2019101).

### Outdoor Air Pollutants and Meteorology Data

According to the monitoring methods described in the previous studies (14, 15, 21), the meteorological factors (temperature and relative humidity) and air pollution data (PM_2.5_, PM_10_, O_3_, NO_2_, and SO_2_) were obtained from open-access government air-quality monitoring stations closed to the participants’ homes. The 24-h average concentrations of PM_2.5_, PM_10_, NO_2_, and SO_2_ as well as the 8-h maximum values of O_3_ were collected as daily exposures. The mean concentrations of air pollution data for 1 day, 1 week, and 1 month before the examination date were recorded for further analysis. Tapered element oscillating microbalance (TEOM) was used to measure the daily concentrations of PM_2.5_ and PM_10_. The daily average concentrations of O_3_ were measured using the non-dispersive ultraviolet fluorescence photometer. The ultraviolet fluorescence and chemiluminescence were applied to measure SO_2_ and NO_2_ levels. According to the distance between the participants’ home and the monitor location, the exposed air pollution data for each patient was obtained from the closest monitoring station. The mean distance between subjects’ homes and their nearest monitor stations was 0.92 ± 0.57 km (range 0.20–2.55 km). Subjects were required to do 3–4 h outdoor activities per day (average) in the corresponding zone. Since the patients were enrolled from the industrial and densely populated areas, the primary sources of PM are the traffic emission, combustion, and sandstorms (22–26). The PM compositions are predominantly organic compound and inorganic salt (nitrate and sulfate).

### Ocular Surface Health Assessment

Individuals’ symptoms were assessed using the OSDI questionnaire (27). Schirmer’s I test (ST), tear meniscus height (TMH), TBUT, corneal fluorescein staining (CFS) score, and meibomian gland (MG) morphology/function of individuals’ right eyes were examined using previously reported methods (28, 29). The CFS score was classified as follows (30): 0 = no staining; 1 = fewer than five dots; 2 = between one and three scores; and 3 = bulk or strip staining. The cornea was divided into four quadrants (superior temporal, inferior temporal, superior nasal, and inferior nasal), and each quadrant was scored separately and summed to obtain the final score. The TMH, TBUT, and MG morphology were recorded using a Keratograph 5 M (OCULUS, Wetzlar, Germany). A four-point grading scale (0–3) was used to grade the area of MG loss (31): 0 (no dropout), 1 (dropout of <1/3rd of the total area), 2 (dropout of 1/3rd to 2/3rd of the total area), and 3 (dropout of >2/3rd of the total area). The MG secretion was graded on a four-point categorical scale (0–3) (32): 0 (clear meibum), 1 (cloudy meibum), 2 (granular meibum), and 3 (inspissated meibum). MG expression was evaluated in five glands on the temporal, central, and nasal eyelids by using the following standard: 0 (all glands expressible), 1 (three to four glands expressible), 2 (one to two glands expressible), and 3 (no glands expressible) (33).

### Tear Film Collection and Cytokine Measurement

Non-irritating tear collection was conducted without anesthesia by using 5-μl capillary pipettes. A plastic head was used to squeeze tears into 0.2-ml Eppendorf tubes, which were immediately frozen at −80°C. The levels of cytokines, such as interleukin (IL)-1 beta (IL-1β), IL-6, IL-8, IL-10, IL-17, tumor necrosis factor-alpha (TNF-α), interferon-gamma (IFN-γ), vascular endothelial growth factor (VEGF), and B-cell activating factor (BAFF), in the undiluted tear samples (at least 50 μl) were measured using a flow cytometer (BD FACS Canto II, Becton Dickinson, Franklin Lakes, NJ, United States) and a bead-array system (BD Cytometric Bead Array system, Becton Dickinson) in accordance with the manufacturer’s instructions. The tear samples were undiluted and each tear volume was inevitably small. Therefore, each sample was measured only once.

### Covariates

Plenty of factors can influence DED (34), such as sex, age, income and education level, hypertension, diabetes mellitus, thyroid disease, rheumatoid arthritis, smoking, season change, temperature, and environmental humidity (14). We considered those factors as covariates, including the laterality of participants’ eyes.

### Statistical Analysis

Participants were divided into four age groups (0–20, 21–40, 41–60, and >60 years), two sex-related groups (male and female), two income level (high and low), two education level (university or higher and high school graduation or less) and two seasonal groups (warm season from April to September, and cold season from January to March and October to December). Continuous variables were presented as mean ± standard deviation (SD). Categorical variables were expressed as frequencies and percentages. A linear mixed model was used to evaluate changes in ocular surface parameters and tear cytokines according to each air pollutant for 1 day, 1 week, and 1 month prior to the examination date. After variables collinearity checking, single-pollutant and multi-pollutant models were developed. Aforementioned covariates were adjusted for both models and got the minimized Akaike Information Criteria (AIC) value. Therefore, the models in this study include all confounding factors. The statistical analysis was performed using SPSS version 23.0 (IBM Corp., Armonk, NY, United States). A *p*-value < 0.05 was considered significant for all comparisons. Multiple comparisons were controlled for by the Bonferroni correction. Since the cytokine concentrations did not show a normal distribution, normality transition was performed before analysis.

## Results

### Demographic Characteristics and Clinical Data

A total of 387 participants were recruited in this study. Detailed demographic characteristics are shown in Table 1. The number of female patients (*n* = 253, 65.4%) was almost twice the number of male patients (*n* = 134, 34.6%). Most patients were aged 21–40 years (*n* = 159, 41.1%) or over 60 years (*n* = 145, 37.5%). The number of patients who visited the hospitals in the warm and cold seasons did not differ significantly. Most patients were classified into severity grading 3 (*n* = 232, 59.9%), followed by grading 2 (*n* = 111, 28.7%), grading 1 (*n* = 35, 9.1%), and grading 4 (*n* = 9, 2.3%). Clinical characteristics and tear cytokines in patients with different severity grades are shown in Table 2. There were significant differences in the ST, TMH, TBUT, CFS score, MG function (expression, secretion, and loss), and VEGF concentrations among grading groups. However, no significant difference was observed in the OSDI score and the concentrations of IL-1β, IL-6, and IL-8.

**TABLE 1:** Demographic characteristics and seasonal distribution of dry eye disease patients.

Characteristics	Number of patients	Percentage (%)
**Sex**		
Male	134	34.6
Female	253	65.4
**Age (years)**		
0–20	4	1.0
21–40	159	41.1
41–60	79	20.4
>61	145	37.5
**Income level**		
High (first, second quartile group)	111	28.7
Low (third, fourth quartile group)	276	71.3
**Education level**		
University or higher	179	46.3
High school graduation or less	208	53.7
Hypertension	91	23.5
Diabetes mellitus	101	26.1
Thyroid disease	65	16.8
Rheumatoid arthritis	58	15.0
Smoking	114	29.5
**Season**		
Warm season	178	46.0
Cold season	209	54.0
**Severity grading**		
1	35	9.1
2	111	28.7
3	232	59.9
4	9	2.3
Total patients	387	100

**TABLE 2:** Clinical characteristics and tear cytokines in patients with different severity grades.

Parameters	Severity grading				*p*
	1 (*n* = 35)	2 (*n* = 111)	3 (*n* = 232)	4 (*n* = 9)	
OSDI (score)^&^	20.25 ± 11.97	22.78 ± 14.28	22.87 ± 11.60	24.73 ± 15.19	0.677
ST (mm)^&^	11.00 ± 0.55	8.26 ± 1.69	4.35 ± 0.59	1.45 ± 0.47	0.000*
TMH (mm)^&^	0.38 ± 0.25	0.24 ± 0.12	0.17 ± 0.06	0.08 ± 0.05	0.000*
TBUT (s)^&^	14.11 ± 2.64	7.15 ± 1.27	3.59 ± 1.02	1.00 ± 0.11	0.000*
CFS (score) ^&^	0.08 ± 0.02	0.33 ± 0.05	0.99 ± 0.22	2.30 ± 1.32	0.001*
**Meibomian gland expression^#^**					
0	20 (57.1%)	18 (16.2%)	18 (7.7%)	0	0.000*
1	10 (28.6%)	26 (23.4%)	70 (34.5%)	2 (22.2%)	
2	5 (14.3%)	51 (46.0%)	106 (45.7%)	3 (33.3%)	
3	0	16 (14.4%)	28 (12.1%)	4 (44.5%)	
**Meibomian gland secretion^#^**					
0	24 (68.6%)	15 (13.5%)	38 (16.4%)	0	0.000*
1	6 (17.1%)	43 (38.8%)	74 (31.9%)	2 (22.2%)	
2	3 (8.6%)	29 (26.1%)	63 (27.1%)	5 (55.6%)	
3	2 (5.7%)	24 (21.6%)	57 (24.6%)	2 (22.2%)	
**Meibomian gland loss^#^**					
0	24 (68.6%)	51 (46.0%)	68 (29.3%)	0	0.000*
1	11 (31.4%)	30 (27.0%)	89 (38.4%)	2 (22.2%)	
2	0	18 (16.2%)	48 (20.7%)	5 (55.6%)	
3	0	12 (10.8%)	27 (11.6%)	2 (22.2%)	
IL-1β (pg/mL)^&^	0.65 ± 1.38	0.68 ± 1.46	1.07 ± 3.00	1.65 ± 6.57	0.800
IL-6 (pg/mL)^&^	0.60 ± 0.81	2.42 ± 4.27	3.00 ± 4.49	5.41 ± 12.49	0.211
IL-8 (pg/mL)^&^	65.00 ± 119.82	82.58 ± 124.70	97.99 ± 159.07	106.59 ± 169.72	0.695
VEGF (pg/mL)^&^	6.99 ± 8.88	40.12 ± 94.13	43.32 ± 62.50	68.49 ± 114.32	0.002*

*OSDI, ocular surface disease index; ST, Schirmer’s I test; TMH, tear meniscus height; TBUT, tear film break-up time; CFS, corneal fluorescein staining; IL-1β, interleukin 1 beta; IL-6, interleukin 6; IL-8, interleukin 8; VEGF, vascular endothelial growth factor.*

*^&^Mean ± standard deviation (SD).*

*^#^Number (percentage).*

*p < 0.05.

### The Effects of Air Pollutants on Ocular Surface in the Single-Pollutant Model

The effects of air pollutants on ocular surface in the single-pollutant model are shown in Table 3. Significant associations were found between increased OSDI scores and higher O_3_ exposures for 1 day, 1 week, and 1 month before the examination {β (1 day/week/month) = 0.414 [95% confidence interval (CI): 0.178–0.528]/0.454 (95% CI: 0.186–0.753)/0.486 (95% CI: 0.164–0.796), *p* = 0.004/0.001/0.000, per 1 ppb increase, respectively}, and higher SO_2_ concentrations for 1 day and 1 week [β (1 day/week) = 0.402 (95% CI: 0.127–0.667)/0.520 (95% CI: 0.084–0.956), *p* = 0.004/0.020, per 1 μg/m^3^ increase, respectively]. As for tear secretion, higher O_3_ exposures for 1 day, 1 week, and 1 month were associated with decreased ST [β (1 day/week/month) = −0.113 (95% CI: −0.158 to −0.032)/−0.133 (95% CI: −0.221 to −0.087)/−0.191 (95% CI: −0.283 to −0.091), *p* = 0.043/0.032/0.003, respectively] and TMH [β (1 day/week/month) = −0.089 (95% CI: −0.161 to −0.006)/−0.166 (95% CI: −0.209 to −0.014)/−0.189 (95% CI: −0.225 to −0.013), all *p* = 0.000]. Higher PM_2.5_ exposure was associated with increased ST for 1 day, 1 week, and 1 month [β (1 day/week/month) = 0.044 (95% CI: −0.038 to 0.127)/0.121 (95% CI: 0.009–0.188)/0.166 (95% CI: 0.014–0.319), *p* = 0.039/0.034/0.033, per 1 μg/m^3^ increase, respectively]; however, with decreased TMH for 1 day [β = −0.087 (95% CI: −0.113 to −0.002), *p* = 0.009] and 1 month [β = −0.014 (95% CI: −0.026 to −0.003), *p* = 0.017]. Higher PM_10_ exposure for 1 day, 1 week, and 1 month were associated with decreased TMH [β (1 day/week/month) = −0.095 (95% CI: −0.158 to −0.003)/ −0.116 (95% CI: −0.201 to −0.011)/−0.210 (95% CI: −0.317 to −0.102), *p* = 0.000/0.007/0.000, per 1 μg/m^3^ increase, respectively]. Adverse associations were found between NO_2_ concentration and ST for 1 month [β = −0.323 (95% CI: −0.492 to −0.154), *p* = 0.000, per 1 μg/m^3^ increase], as well as TMH for 1 day, 1 week, and 1 month [β (1 day/week/month) = −0.011 (95% CI: −0.016 to −0.006)/−0.019 (95% CI: −0.028 to −0.010)/−0.034 (95% CI: −0.044 to −0.025), all *p* = 0.000, respectively]. Adverse associations were found between TBUT and various air pollutants, such as PM_2.5_, PM_10_, O_3_, and SO_2_. Additionally, increased CFS scores were associated with higher PM_2.5_, PM_10_, O_3_, SO_2_, and NO_2_ exposures.

**TABLE 3:** Effects of air pollutants on ocular surface using single-pollutant models.

	PM_2.5_ (per 1 μ g/m^3^)		PM_10_ (per 1 μ g/m^3^)		O_3_ (per 1 ppb increase)		SO_2_ (per 1 μ g/m^3^)		NO_2_ (per 1 μ g/m^3^)	
	Estimate (95% CI)	*p*	Estimate (95% CI)	*p*	Estimate (95% CI)	*p*	Estimate (95% CI)	*p*	Estimate (95% CI)	*p*
**OSDI**										
1 day	−0.075 (−0.276 to 0.125)	0.459	−0.047 (−0.139 to 0.045)	0.317	**0.414 (0.178 to 0.528)** **	**0.004**	**0.402 (0.127 to 0.677)** **	**0.004**	0.101 (−0.096 to 0.298)	0.314
1 week	0.209 (−0.092 to 0.511)	0.172	0.147 (0.021 to 0.314)	0.086	**0.454 (0.186 to 0.753)** **	**0.001**	**0.520 (0.084 to 0.956)** **	**0.020**	0.279 (0.034 to 0.591)	0.080
1 month	0.330 (0.122 to 0.783)	0.152	0.109 (−0.180 to 0.398)	0.459	**0.486 (0.164 to 0.796)** **	**0.000**	0.051 (−0.463 to 0.565)	0.844	−0.121 (−0.483 to 0.242)	0.513
**ST**										
1 day	**0.044 (−0.038 to 0.127)** *	**0.039**	−0.006 (−0.048 to 0.036)	0.782	**−0.113 (−0.158 to −0.032)** *	**0.043**	0.068 (−0.064 to 0.200)	0.313	−0.007 (−0.093 to 0.079)	0.868
1 week	**0.121 (0.009 to 0.233)** *	**0.034**	0.032 (0.008 to 0.111)	0.434	**−0.133 (−0.221 to −0.087)** *	**0.032**	0.015 (−0.276 to 0.307)	0.917	−0.058 (−0.212 to 0.097)	0.463
1 month	**0.166 (0.014 to 0.319)** *	**0.033**	0.093 (−0.041 to 0.277)	0.173	**−0.191 (−0.283 to −0.091)** **	**0.003**	0.181 (−0.071 to 0.433)	0.818	**−0.323 (−0.492 to −0.154)** **	**0.000**
**TMH**										
1 day	**−0.087 (−0.113 to −0.002)** **	**0.009**	**−0.095 (−0.158 to −0.003)** **	**0.000**	**−0.089 (−0.161 to −0.006)** **	**0.000**	−0.001 (−0.009 to 0.007)	0.791	**−0.011 (−0.016 to −0.006)** **	**0.000**
1 week	0.006 (−0.002 to 0.014)	0.156	**−0.116 (−0.201 to −0.011)** **	**0.007**	**−0.166 (−0.209 to −0.014)** **	**0.000**	0.011 (0.000 to 0.023)	0.059	**−0.019 (−0.028 to −0.010)** **	**0.000**
1 month	**−0.014 (−0.026 to −0.003)** *	**0.017**	**−0.210 (−0.317 to −0.102)** *	**0.012**	**−0.189 (−0.225 to −0.013)** **	**0.000**	0.016 (0.003 to 0.029)*	0.059	**−0.034 (−0.044 to −0.025)** **	**0.000**
**TBUT**										
1 day	**−0.074 (−0.112 to −0.036)** **	**0.000**	0.006 (−0.012 to 0.023)	0.516	**−0.020 (−0.040 to −0.001)** *	**0.041**	**−0.127 (−0.175 to −0.078)** **	**0.000**	0.034 (−0.003 to 0.071)	0.068
1 week	**−0.066 (−0.113 to −0.018)** **	**0.007**	**−0.028 (−0.042 to −0.018)** *	**0.034**	0.022 (−0.001 to 0.046)	0.065	**−0.272 (−0.350 to −0.193)** **	**0.000**	0.048 (−0.008 to 0.104)	0.093
1 month	**−0.100 (−0.183 to −0.016)** *	**0.019**	**−0.029 (−0.036 to −0.011)** *	**0.010**	−0.031 (−0.074 to 0.011)	0.148	**0.206 (−0.301 to −0.111)** **	**0.000**	−0.040 (−0.107 to 0.027)	0.240
**CFS**										
1 day	−0.001 (−0.027 to 0.024)	0.925	**0.028 (0.017 to 0.040)** **	**0.000**	**0.018 (0.005 to 0.031)** **	**0.000**	**0.089 (0.054 to 0.123)** **	**0.000**	**0.051 (0.026 to 0.076)** **	**0.000**
1 week	**0.107 (0.060 to 0.134)** **	**0.000**	**0.045 (0.028 to 0.073)** **	**0.000**	0.009 (−0.007 to 0.025)	0.278	**0.124 (0.071 to 0.178)** **	**0.000**	0.019 (−0.019 to 0.058)	0.318
1 month	**0.150 (0.093 to 0.208)** **	**0.000**	**0.073 (0.037 to 0.110)** **	**0.000**	0.018 (−0.012 to 0.047)	0.235	**0.122 (0.057 to 0.188)** **	**0.000**	**0.104 (0.058 to 0.150)** **	**0.000**
**MG expression**										
1 day	0.009 (−0.002 to 0.019)	0.119	0.003 (−0.002 to 0.008)	0.206	0.009 (0.003 to 0.015)	0.052	0.017 (0.002 to 0.032)	0.053	0.004 (−0.007 to 0.014)	0.477
1 week	0.011 (−0.006 to 0.027)	0.203	0.013 (0.003 to 0.029)	0.103	0.001 (−0.006 to 0.009)	0.707	0.017 (−0.007 to 0.040)	0.168	−0.009 (−0.026 to 0.008)	0.295
1 month	**0.035 (0.011 to 0.060)** **	**0.005**	**0.019 (0.009 to 0.021)** *	**0.045**	**0.015 (0.002 to 0.028)** **	**0.020**	0.003 (−0.025 to 0.032)	0.808	**0.022 (0.002 to 0.042)** *	**0.033**
**MG secretion**										
1 day	−0.003 (−0.016 to 0.009)	0.593	0.001 (−0.005 to 0.007)	0.727	0.005 (−0.002 to 0.011)	0.172	0.021 (0.004 to 0.038)	0.068	**0.023 (0.010 to 0.035)** **	**0.000**
1 week	0.001 (−0.017 to 0.019)	0.907	−0.003 (−0.013 to 0.008)	0.617	0.007 (−0.001 to 0.015)	0.098	0.011 (−0.015 to 0.038)	0.396	**0.039 (0.020 to 0.058)** **	**0.000**
1 month	0.013 (−0.014 to 0.040)	0.336	0.003 (−0.015 to 0.020)	0.758	**0.068 (0.046 to 0.089)** **	**0.000**	−0.022 (−0.053 to 0.008)	0.153	**0.068 (0.046 to 0.089)** **	**0.000**
**MG loss**										
1 day	**0.021 (0.009 to 0.034)** **	**0.001**	**0.013 (0.001 to 0.024)** *	**0.028**	**0.016 (0.010 to 0.023)** **	**0.000**	0.003 (0.002 to 0.014)	0.720	0.007 (−0.005 to 0.020)	0.250
1 week	**0.012 (−0.005 to 0.029)** *	**0.011**	**0.015 (0.006 to 0.024)** **	**0.002**	**0.019 (0.007 to 0.026)** *	**0.023**	−0.004 (−0.029 to 0.020)	0.725	0.006 (−0.011 to 0.024)	0.486
1 month	**0.075 (0.051 to 0.100)** **	**0.000**	**0.024 (0.009 to 0.040)** **	**0.003**	**0.025 (0.012 to 0.037)** **	**0.000**	−0.002 (−0.030 to 0.026)	0.899	**0.025 (0.006 to 0.045)** *	**0.012**
**IL-1β**										
1 day	−0.007 (−0.015 to 0.001)	0.093	−0.002 (−0.006 to 0.001)	0.190	0.001 (0.000 to 0.005)	0.569	−0.006 (−0.017 to 0.005)	0.266	0.006 (−0.002 to 0.014)	0.130
1 week	**0.011 (−0.001 to 0.021)** *	**0.025**	**0.009 (0.002 to 0.015)** *	**0.010**	0.005 (0.002 to 0.013)	0.169	0.004 (−0.014 to 0.021)	0.674	0.006 (−0.008 to 0.021)	0.406
1 month	0.046 (−0.004 to 0.096)	0.069	0.010 (0.009 to 0.029)	0.313	0.007 (−0.005 to 0.020)	0.255	0.011 (−0.008 to 0.030)	0.254	−0.009 (−0.027 to 0.009)	0.336
**IL-6**										
1 day	−0.013 (−0.026 to 0.001)	0.062	**0.015 (0.001 to 0.031)** *	**0.042**	**0.018 (−0.003 to 0.038)** *	**0.040**	−0.002 (−0.019 to 0.015)	0.819	**0.016 (0.003 to 0.029)** *	**0.015**
1 week	0.002 (−0.033 to 0.029)	0.896	0.009 (−0.020 to 0.019)	0.103	0.005 (−0.017 to 0.008)	0.468	0.000 (−0.029 to 0.028)	0.985	**0.026 (0.002 to 0.049)** *	**0.034**
1 month	0.042 (−0.038 to 0.122)	0.300	0.001 (−0.004 to 0.007)	0.604	−0.004 (−0.011 to 0.003)	0.282	0.005 (−0.026 to 0.036)	0.753	**0.035 (0.006 to 0.064)** *	**0.019**
**IL-8**										
1 day	**0.018 (0.004 to 0.031)** **	**0.009**	**0.013 (0.000 to 0.025)** *	**0.045**	−0.006 (−0.013 to 0.001)	0.070	0.005 (−0.012 to 0.022)	0.542	**0.013 (0.000 to 0.025)** *	**0.044**
1 week	0.017 (−0.015 to 0.050)	0.291	0.001 (−0.010 to 0.012)	0.843	0.003 (−0.010 to 0.016)	0.614	0.009 (−0.021 to 0.038)	0.556	−0.001 (−0.025 to 0.024)	0.946
1 month	0.028 (−0.055 to 0.111)	0.509	−0.016 (−0.032 to 0.001)	0.065	0.004 (−0.017 to 0.025)	0.715	0.015 (−0.017 to 0.047)	0.372	0.015 (−0.015 to 0.045)	0.321
**VEGF**										
1 day	**0.014 (0.002 to 0.025)**	**0.018**	**0.011 (0.000 to 0.022)** *	**0.043**	0.006 (0.000 to 0.012)	0.061	−0.002 (−0.017 to 0.012)	0.762	0.010 (−0.001 to 0.021)	0.073
1 week	0.003 (−0.024 to 0.030)	0.821	−0.001 (−0.011 to 0.008)	0.807	−0.003 (−0.014 to 0.008)	0.555	−0.014 (−0.039 to 0.011)	0.259	−0.015 (−0.035 to 0.006)	0.163
1 month	0.057 (−0.012 to 0.127)	0.107	0.012 (−0.001 to 0.026)	0.080	0.006 (−0.012 to 0.023)	0.532	−0.001 (−0.028 to 0.026)	0.951	−0.020 (−0.045 to 0.005)	0.119

PM, particulate matter; O3, ozone; SO2, sulfur dioxide; NO2, nitrogen dioxide; CI, confidence interval; OSDI, ocular surface disease index; ST, Schirmer’s I test; TMH, tear meniscus height; TBUT, tear film break-up time; CFS, corneal fluorescein staining; MG, meibomian gland; IL-1β, interleukin 1 beta; IL-6, interleukin 6; IL-8, interleukin 8; VEGF, vascular endothelial growth factor. The Estimate (95% CI) and p value are shown for all significant associations in bold. *p < 0.05; **p < 0.01.

Exposure to air pollution for 1 month had a greater effect on MG, such as MG expression and PM_2.5_ [β = 0.035 (95% CI: 0.011–0.060), *p* = 0.005], PM_10_ [β = 0.019 (95% CI: 0.009–0.021), *p* = 0.045], O_3_ [β = 0.015 (95% CI: 0.002–0.028), *p* = 0.020] and NO_2_ [β = 0.022 (95% CI: 0.002–0.042), *p* = 0.033]; MG secretion and O_3_ [β = 0.068 (95% CI: 0.046–0.089), *p* = 0.000], and NO_2_ [β = 0.068 (95% CI: 0.046–0.089), *p* = 0.000]; MG loss and NO_2_ [β = 0.025 (95% CI: 0.006–0.045), *p* = 0.012], PM_2.5_ [β = 0.075 (95% CI: 0.051–0.100), *p* = 0.000], PM_10_ [β = 0.024 (95% CI: 0.009–0.040), *p* = 0.003], and O_3_ [β = 0.025 (95% CI: 0.012–0.037), *p* = 0.000].

Exposure to air pollution for 1 day had a greater effect on tear cytokines, such as PM_2.5_ and IL-8 [β = 0.018 (95% CI: 0.004–0.031), *p* = 0.009], and VEGF [β = 0.014 (95% CI: 0.002–0.025), *p* = 0.018]; PM_10_ and IL-6 [β = 0.015 (95% CI: 0.001–0.031), *p* = 0.042], IL-8 [β = 0.013 (95% CI: 0.000–0.025), *p* = 0.045] and VEGF [β = 0.011 (95% CI: 0.000–0.022), *p* = 0.043]; O_3_ and IL-6 [β = 0.018 (95% CI: −0.003 to 0.038), *p* = 0.040]; NO_2_ and IL-8 [β = 0.013 (95% CI: 0.000–0.025), *p* = 0.044]. Higher PM exposure for 1 week was associated with IL-1β [PM_2.5_: β = 0.011 (95% CI: −0.001 to 0.021), *p* = 0.025; PM_10_: 0.009 (95% CI: 0.002–0.015), *p* = 0.010]. Higher NO_2_ exposure for 1 day, 1 week, and 1 month were associated with IL-6 [β (1 day/week/month) = 0.016 (95% CI: 0.003–0.029)/0.026 (95% CI: 0.002–0.049)/0.035 (95% CI: 0.006–0.064), *p* = 0.015/0.034/0.019, respectively]. There was no association between SO_2_ exposure and tear cytokines.

### The Effects of Air Pollutants on Ocular Surface in the Multi-Pollutant Model

The effects of air pollutants on ocular surface in the multi-pollutant model are shown in Table 4. Multicollinearity analyses among all air pollutants were assessed to ensure the variance inflation factors less than 10 in this model. Higher O_3_ exposures for 1 day, 1 week, and 1 month were associated with an increased OSDI score as well as decreased ST and TMH [OSDI: β (1 day/week/month) = 0.403 (95% CI: 0.229–0.523)/0.471 (95% CI: 0.252–0.693)/0.468 (95% CI: 0.215–0.732), *p* = 0.020/0.008/0.040; ST: β (1 day/week/month) = −0.117 (95% CI: −0.149 to −0.008)/−0.125 (95% CI: −0.178 to −0.068)/−0.114 (95% CI: −0.200 to −0.029), *p* = 0.033/0.029/0.009; TMH: β (1 day/week/month) = −0.075 (95% CI: −0.127 to −0.010)/−0.136 (95% CI: −0.209 to −0.053)/−0.118 (95% CI: −0.223 to −0.022), all *p* = 0.000]. Higher SO_2_ exposures were associated with increased OSDI and CFS score, as well as decreased TBUT [OSDI: β (1 day/week) = 0.437 (95% CI: 0.193–0.680)/0.470 (95% CI: 0.040–0.901), *p* = 0.000/0.032; CFS: β (1 day/week/month) = 0.089 (95% CI: 0.054–0.123)/0.106 (95% CI: 0.059–0.154)/0.073 (95% CI: 0.007–0.138), *p* = 0.000/0.000/0.029; TBUT: β (1 day/week/month) = −0.122 (95% CI: −0.170 to −0.073)/ −0.293 (95% CI: −0.363 to −0.224)/−0.241 (95% CI: −0.307 to −0.174), all *p* = 0.000]. Unlike in the single-pollutant model, higher PM_2.5_ concentrations for 1 week and 1 month were associated with an increased OSDI score [β (1 week/month) = 0.229 (95% CI: 0.035–0.424)/0.211 (95% CI: 0.160–0.583), *p* = 0.021/0.014, respectively]. Moreover, higher PM_2.5_ concentration was associated with increased ST for 1 day [β = 0.246 (95% CI: 0.106–0.328), *p* = 0.029] and 1 week [β = 0.202 (95% CI: 0.150–0.365), *p* = 0.046]; but decreased TMH for 1 day [β = −0.086 (95% CI: −0.112 to −0.010), *p* = 0.029], 1 week [β = −0.043 (95% CI: −0.085 to 0.021), *p* = 0.042], and 1 month [β = −0.023 (95% CI: −0.033 to −0.014), *p* = 0.000]. Higher PM_10_ exposure for 1 month was also associated with decreased TMH [β = −0.015 (95% CI: −0.021 to −0.009), *p* = 0.000], but not associated with OSDI and ST. Similarity, air pollutants showed adverse associations with TBUT and positive effects on CFS, and those effects were more apparently for 1-week exposure. However, exposure to PM, O_3_, and NO_2_ for 1 month showed higher effects on MG function.

**TABLE 4:** Effects of air pollutants on ocular surface using multi-pollutant models.

	PM_2.5_ (per 1 μ g/m^3^)		PM_10_ (per 1 μ g/m^3^)		O_3_ (per 1 ppb increase)		SO_2_ (per 1 μ g/m^3^)		NO_2_ (per 1 μ g/m^3^)	
	Estimate (95% CI)	*p*	Estimate (95% CI)	*p*	Estimate (95% CI)	*p*	Estimate (95% CI)	*p*	Estimate (95% CI)	*p*
**OSDI**										
1 day	0.023 (−0.012 to 0.059)	0.197	−0.023 (−0.054 to 0.008)	0.147	**0.403 (0.229 to 0.523)** *	**0.020**	**0.437 (0.193 to 0.680)** **	**0.000**	0.006 (−0.003 to 0.112)	0.925
1 week	**0.229 (0.035 to 0.424)** *	**0.021**	0.117 (0.045 to 0.279)	0.156	**0.471 (0.252 to 0.693)** **	**0.008**	**0.470 (0.040 to 0.901)** **	**0.032**	0.262 (0.050 to 0.574)	0.099
1 month	**0.211 (0.160 to 0.583)** *	**0.014**	0.017 (−0.216 to 0.249)	0.887	**0.468 (0.215 to 0.732)** *	**0.040**	0.160 (−0.332 to 0.653)	0.522	−0.091 (−0.452 to 0.270)	0.620
**ST**										
1 day	**0.246 (0.106 to 0.328)** *	**0.029**	−0.009 (−0.051 to 0.033)	0.680	**−0.117 (−0.149 to −0.008)** *	**0.033**	0.076 (−0.044 to 0.195)	0.213	−0.006 (−0.083 to 0.067)	0.871
1 week	**0.202 (0.150 to 0.365)** *	**0.046**	0.039 (−0.039 to 0.117)	0.326	**−0.125 (−0.178 to −0.068)** *	**0.029**	0.012 (−0.279 to 0.303)	0.935	−0.077 (−0.223 to 0.070)	0.302
1 month	−0.011 (−0.152 to 0.130)	0.880	0.048 (−0.0531 to 0.150)	0.349	**−0.114 (−0.200 to −0.029)** **	**0.009**	0.262 (0.038 to 0.486)	0.052	**−0.299 (−0.465 to −0.134)** **	**0.000**
**TMH**										
1 day	**−0.086 (−0.112 to −0.010)** *	**0.029**	−0.005 (−0.008 to −0.003)	0.051	**−0.075 (−0.127 to −0.010)** **	**0.000**	0.004 (−0.003 to 0.011)	0.279	**−0.015 (−0.019 to −0.010)** **	**0.000**
1 week	**−0.043 (−0.085 to 0.021)** *	**0.042**	−0.005 (−0.010 to −0.001)	0.066	**−0.136 (−0.209 to −0.053)** **	**0.000**	0.010 (−0.002 to 0.022)	0.093	**−0.019 (−0.027 to −0.010)** **	**0.000**
1 month	**−0.023 (−0.033 to −0.014)** *	**0.000**	**−0.015 (−0.021 to −0.009)** **	**0.000**	**−0.118 (−0.223 to −0.022)** **	**0.000**	0.021 (0.008 to 0.034)	0.052	**−0.033 (−0.042 to −0.023)** **	**0.000**
**TBUT**										
1 day	**−0.075 (−0.112 to −0.038)** **	**0.000**	0.009 (−0.009 to 0.026)	0.325	**−0.024 (−0.039 to −0.010)** **	**0.001**	**−0.122 (−0.170 to −0.073)** **	**0.000**	0.032 (−0.004 to 0.069)	0.081
1 week	**−0.079 (−0.148 to −0.011)** *	**0.023**	**−0.024 (−0.043 to −0.016)** *	**0.012**	0.013 (−0.007 to 0.033)	0.197	**−0.293 (−0.363 to −0.224)** **	**0.000**	0.049 (−0.007 to 0.105)	0.086
1 month	**−0.074 (−0.141 to −0.007)** *	**0.031**	−0.010 (−0.054 to 0.033)	0.634	−0.041 (−0.079 to 0.003)	0.053	**0.241 (0.174 to 0.307)** **	**0.000**	−0.044 (−0.110 to 0.023)	0.199
**CFS**										
1 day	−0.007 (−0.033 to 0.019)	0.608	**0.047 (0.030 to 0.064)** **	**0.000**	**0.018 (0.008 to 0.029)** **	**0.001**	**0.089 (0.054 to 0.123)** **	**0.000**	**0.051 (0.029 to 0.072)** **	**0.000**
1 week	**0.090 (0.054 to 0.126)** **	**0.000**	**0.100 (0.052 to 0.148)** **	**0.000**	**0.044 (0.018 to 0.071)** **	**0.001**	**0.106 (0.059 to 0.154)** **	**0.000**	0.027 (−0.013 to 0.067)	0.181
1 month	**0.082 (0.034 to 0.129)** **	**0.001**	**0.055 (0.025 to 0.084)** **	**0.000**	**0.014 (0.001 to 0.029)** *	**0.045**	**0.073 (0.007 to 0.138)** *	**0.029**	**0.133 (0.067 to 0.160)** **	**0.000**
**MG expression**										
1 day	0.008 (−0.003 to 0.019)	0.148	0.003 (−0.002 to 0.008)	0.261	0.007 (0.002 to 0.011)	0.052	0.014 (0.000 to 0.028)	0.068	0.003 (−0.008 to 0.013)	0.597
1 week	0.006 (−0.010 to 0.022)	0.432	−0.008 (−0.017 to 0.001)	0.083	−0.001 (−0.007 to 0.005)	0.698	0.014 (−0.009 to 0.037)	0.236	−0.008 (−0.025 to 0.009)	0.341
1 month	**0.023 (0.003 to 0.043)** **	**0.003**	**0.021 (0.000 to 0.041)** **	**0.047**	**0.020 (0.009 to 0.031)** **	**0.001**	0.013 (0.007 to 0.033)	0.191	**0.023 (0.004 to 0.043)** *	**0.021**
**MG secretion**										
1 day	−0.004 (−0.016 to 0.009)	0.569	0.001 (−0.005 to 0.007)	0.688	0.006 (0.000 to 0.011)	0.056	0.020 (0.003 to 0.038)	0.060	**0.021 (0.010 to 0.032)** **	**0.000**
1 week	0.000 (−0.018 to 0.017)	0.991	−0.002 (−0.012 to 0.007)	0.626	0.008 (0.000 to 0.015)	0.054	0.008 (−0.015 to 0.032)	0.486	**0.038 (0.020 to 0.057)** **	**0.000**
1 month	**0.027 (0.005 to 0.049)** *	**0.018**	0.008 (−0.006 to 0.022)	0.273	**0.027 (0.014 to 0.039)** **	**0.000**	−0.019 (−0.040 to 0.003)	0.084	**0.067 (0.046 to 0.089)** **	**0.000**
**MG loss**										
1 day	**0.021 (0.009 to 0.034)** **	**0.000**	0.000 (−0.006 to 0.006)	0.909	**0.015 (0.010 to 0.020)** **	**0.000**	−0.004 (−0.021 to 0.012)	0.604	0.008 (−0.007 to 0.020)	0.271
1 week	**0.026 (0.015 to 0.037)** **	**0.000**	0.004 (−0.009 to 0.018)	0.524	**0.013 (0.006 to 0.020)** **	**0.000**	−0.007 (−0.031 to 0.017)	0.560	0.007 (−0.010 to 0.025)	0.422
1 month	**0.053 (0.032 to 0.073)** **	**0.000**	**0.013 (0.004 to 0.022)** **	**0.004**	**0.033 (0.022 to 0.045)** **	**0.000**	−0.033 (−0.053 to 0.013)	0.101	**0.028 (0.008 to 0.048)** *	**0.005**
**IL−1β**										
1 day	−0.005 (−0.013 to 0.002)	0.175	−0.001 (−0.005 to 0.002)	0.392	0.001 (−0.002 to 0.004)	0.606	−0.004 (−0.015 to 0.006)	0.404	0.007 (−0.001 to 0.015)	0.070
1 week	**0.009 (0.001 to 0.018)** *	**0.033**	**0.008 (0.001 to 0.014)** *	**0.017**	0.001 (−0.005 to 0.007)	0.687	0.005 (−0.012 to 0.022)	0.555	0.002 (−0.010 to 0.014)	0.737
1 month	0.017 (−0.026 to 0.060)	0.437	0.007 (0.002 to 0.015)	0.124	0.007 (−0.006 to 0.019)	0.302	0.003 (−0.014 to 0.020)	0.761	0.004 (−0.009 to 0.016)	0.571
**IL-6**										
1 day	**0.014 (0.001 to 0.027)** *	**0.035**	**0.019 (0.006 to 0.033)** **	**0.006**	**0.005 (0.000 to 0.010)** *	**0.041**	−0.003 (−0.020 to 0.014)	0.721	**0.015 (0.003 to 0.028)** *	**0.017**
1 week	0.006 (−0.023 to 0.036)	0.670	−0.009 (−0.019 to 0.001)	0.092	0.002 (−0.007 to 0.012)	0.661	0.000 (−0.028 to 0.028)	0.999	**0.025 (0.005 to 0.044)** *	**0.014**
1 month	0.081 (0.011 to 0.150)	0.053	0.003 (−0.002 to 0.008)	0.271	0.018 (−0.002 to 0.039)	0.078	0.013 (−0.014 to 0.040)	0.356	**0.045 (0.018 to 0.072)** **	**0.001**
**IL-8**										
1 day	**0.016 (0.003 to 0.029)** **	**0.013**	**0.008 (0.001 to 0.016)** *	**0.034**	−0.004 (−0.009 to 0.000)	0.078	0.007 (−0.010 to 0.024)	0.415	**0.014 (0.001 to 0.026)** *	**0.033**
1 week	0.018 (−0.012 to 0.049)	0.234	0.003 (−0.008 to 0.014)	0.565	0.004 (−0.006 to 0.014)	0.405	0.007 (−0.018 to 0.033)	0.571	0.006 (−0.014 to 0.027)	0.544
1 month	−0.012 (−0.084 to 0.060)	0.749	−0.013 (−0.027 to 0.001)	0.057	0.003 (−0.018 to 0.024)	0.775	−0.009 (−0.029 to 0.011)	0.392	**0.023 (0.002 to 0.043)**	**0.029**
**VEGF**										
1 day	**0.011 (0.000 to 0.022)**	**0.044**	−0.002 (−0.007 to 0.003)	0.396	0.003 (0.001 to 0.007)	0.169	0.001 (−0.014 to 0.015)	0.965	0.012 (0.001 to 0.023)	0.053
1 week	0.004 (−0.021 to 0.030)	0.745	−0.002 (−0.010 to 0.007)	0.735	−0.004 (−0.011 to 0.002)	0.194	−0.015 (−0.039 to 0.010)	0.237	−0.013 (−0.030 to 0.004)	0.129
1 month	0.026 (−0.035 to 0.086)	0.401	0.007 (−0.005 to 0.019)	0.279	0.006 (−0.012 to 0.023)	0.532	−0.019 (−0.037 to −0.002)	0.126	−0.011 (−0.035 to 0.012)	0.338

PM, particulate matter; O3, ozone; SO2, sulfur dioxide; NO2, nitrogen dioxide; CI, confidence interval; OSDI, ocular surface disease index; ST, Schirmer’s I test; TMH, tear meniscus height; TBUT, tear film break-up time; CFS, corneal fluorescein staining; MG, meibomian gland; IL-1β, interleukin 1 beta; IL-6, interleukin 6; IL-8, interleukin 8; VEGF, vascular endothelial growth factor. The Estimate (95% CI) and p value are shown for all significant associations in bold. *p < 0.05; **p < 0.01.

Exposure to PM and O_3_ for 1 day had greater effects on tear cytokines, such as PM_2.5_ and IL-6 [β = 0.014 (95% CI: 0.001–0.027), *p* = 0.035], IL-8 [β = 0.016 (95% CI: 0.003–0.029), *p* = 0.013], VEGF [β = 0.011 (95% CI: 0.000–0.022), *p* = 0.044]; PM_10_ and IL-6 [β = 0.019 (95% CI: 0.006–0.033), *p* = 0.006], IL-8 [β = 0.008 (95% CI: 0.001–0.016), *p* = 0.034]; O_3_ and IL-6 [β = 0.005 (95% CI: 0.000–0.010), *p* = 0.041]. Higher PM exposures were associated with increased IL-1β concentration for 1 week [PM_2.5_: β = 0.009 (95% CI: 0.001–0.018), *p* = 0.033; PM_10_: β = 0.008 (95% CI: 0.001–0.014), *p* = 0.017]. However, exposure to NO_2_ for 1 month had greater effects on tear cytokines, such as NO_2_ and IL-6 [β = 0.045 (95% CI: 0.018–0.072), *p* = 0.001], NO_2_ and IL-8 [β = 0.023 (95% CI: 0.002–0.043), *p* = 0.029].

## Discussion

The multicenter prospective cohort study found that higher PM_2.5_, O_3_, and SO_2_ exposures could increase ocular surface discomfort, aggravate tear film stability, and deteriorate ocular surface damage. Increased PM_10_ concentration also led tear film instability and ocular injury, however, it was not associated with an increased OSDI score. Increased O_3_ and NO_2_ concentrations decreased tear secretion, higher PM_2.5_ level increased ST while decreased TMH. Exposure to high levels of air pollutants (except SO_2_) also aggravated meibomian gland dysfunction (MGD) and upregulated tear inflammatory cytokine concentrations. Interestingly, the time windows of different air pollutants exposure on different DED parameters were diverse. Exposure to air pollutants for 1 week before the examination had the greatest effects on the discomforts and clinical data of DED, while exposure to air pollution for 1 month and 1 day showed more apparently influences on MG functions and tear cytokines, respectively.

The PM has become one of the crucial air pollutants and can result in various diseases of human beings (21, 35). The development in industrialization and urbanization has led to air pollution as the biggest social issue in China recently, and PM levels in China often exceeded normal range and reached “bad” level according to the WHO air quality guidelines. The constituents of PM are diverse and complex, mainly such as polyaromatic hydrocarbons, nitrate, sulfate, organic carbon, heavy metals, and among others (21). Since the continuously changed atmospheric chemistry and weather conditions in different time and locations, and the complex interactions with other air pollutants, the PM compositions are diverse and can play various roles on the ocular surface (21). The patients in the present study were enrolled from the industrial and densely populated areas, the predominant compositions of PM are organic compound, nitrate, and sulfate. It may be hard for us to determine the specific effects of PM on the ocular surface because of the heterogeneity. However, several confounding factors including humidity and season have been adjusted and consistent results were found both in the single and multi-pollutant models. The oxidative stress has been proved to be a main harmful effect of PM (17, 18). Increased PM_2.5_ and PM_10_ exposure on the ocular surface could cause tear film instability and homeostasis imbalance, then lead to ocular surface damage (17, 18). Higher PM concentrations also could impair corneal epithelial cell and conjunctival goblet cells, as well as increase the release of pro-inflammatory factors, including TNF-α and phosphorylated NF-κB in mice (17, 18). Those results were consistent with our findings. In the present study, high PM exposures were associated with the increased tear film instability and ocular surface damage. High PM_2.5_ exposures were associated with more serious dry eye complaints and increased ST. Interestingly, PM could stimulate the tear production (increased ST) but could not remain tears on the ocular surface (decreased TMH), this might also be attributed to the poor tear film stability. Increased PM_2.5_ concentration was closely associated with a decreased TBUT in both the single- and multi-pollutant models compared to PM_10_. Moreover, increased PM_2.5_ concentration was associated with an increased OSDI score. These diversities may be because of the differences in particle sizes. Among all the coarse particles, PM_10_ is the largest one. The large particle size may influence the contact areas with the tear film and lead to a lower effect than PM_2.5_. Compared to PM_10_, PM_2.5_ may adsorb more toxic materials and elicit greater toxicity since the much wider available surface areas.

Similar to the PM, NO_2_ is considered as combustion-derived pollutant from vehicular emissions and biomass burning (36, 37). Several studies have demonstrated the association between conjunctival goblet cell density and NO_2_ level (36, 37). Mucins, which mainly produced from goblet cells, play a key role in keeping tear film stability and ocular surface homeostasis, such as removal of pathogens, allergens and debris, lubrication, and antimicrobial properties (38, 39). Gipson et al. found that increased mucin levels were associated with DED presentation (38). Actually, the excess mucin production is a self-preservation mechanism in humans to defend ocular surface irritation and early stage inflammatory (38, 40). Those funding were consistent with our results that higher NO_2_ concentrations increased the ocular surface damage, impaired the MG function and upregulated the pro-inflammatory factors. However, there was no associations with OSDI scores and TBUT, suggesting the appearance of a compensatory mechanism to avoid dry eye symptoms and keep tear film balance (41). There may be some adaptive responses during continued exposure to air pollution. And though some unknown pathways, increased goblet cell density and mucin levels could remain tears and maintain tear film homeostasis, therefore, patients remain symptom-free temporarily. Additionally, exposure to NO_2_ for 1 month had the greater effects on ocular surface (including MG and cytokines) than exposure for 1 day or 1 week. The damage to the ocular surface was cumulative over time, suggesting that compensatory mechanism may only work within a certain threshold, and long-term exposures causing lasting damage. A study also found the conjunctivitis outpatient visit was small after exposure to NO_2_ immediately but the odds were increasing with time (42).

Epidemiological studies found SO_2_ was derived from the combustion of sulfur-containing fossil fuels of motor vehicles and various industries (43). Exposure to SO_2_ contributes to high morbidity and mortality worldwide (43, 44). Eye sensitivity and irritation were found associated with high SO_2_ exposures (45). Saha et al. suggested that tear film was vulnerable when exposure to combustion products in ambient air (46). Those results were consisted with our findings that increased ground-level SO_2_ concentrations increased ocular discomforts and tear secretions (ST), decreased the TBUT and caused ocular surface damage. The balance and dynamics of tear film are influenced by many factors, such as tear generation and evaporation, eyelid motion, surface tension, and polar lipid of the tear film (47). Tear film can evenly diffuse on the ocular surface because of a reduced air-fluid interface tension (47, 48). As the first physical and chemical barrier, the outermost lipid layer of the precorneal tear film may be influenced by the combustion particulates (PM, NO_2_, and SO_2_) which repeated contact on the air-fluid surface though oxidative damage or other mechanisms, resulting in an increased surface energy. Moreover, there is a negative correlation between TBUT and surface tension (48). As a consequence, decreased TBUT may be associated with higher PM and SO_2_ concentrations.

As a powerful oxidant, Ozone has been reported to be associated with various adverse health effects and even increased the mortality rates (49). The previous studies have shown that the O_3_ exposure was associated with DED. Hwang et al. found that DED symptoms and diagnosis were associated with higher O_3_ exposures in Korea (14). Moreover, Kim et al., demonstrated that higher O_3_ concentrations were associated with increased OSDI scores and decreased tear secretion in DED patients (21). Additionally, Lee et al., reported that O_3_ could upregulate tear inflammatory cytokine levels (IL-1β, IL-6, and IL-17) and decrease conjunctival goblet cell density in mouse models, therefore, resulting in ocular surface discomfort and inflammation (50, 51). This present study also showed that high O_3_ concentrations increased ocular discomfort, decreased tear secretion (both ST and TMH), impaired tear film stability, aggravated ocular surface damage and upregulated tear inflammatory cytokine levels (IL-6). The O_3_ concentration was also associated with MGD, especially in 1 month. It may be based on its ability to produce reactive oxygen species and induce pro-inflammatory cytokines. Also, O_3_ can cause injury to cellular proteins and lipids and the damage may accumulate over time. Importantly, ozone is an atmospheric trace gas with its molecule much smaller than a protein or lipid (43). Therefore, it may approach the ocular surface, such as cornea, lacrimal glands, and MGs, decrease tear secretions and induce ocular surface inflammation (21). The effects of O_3_ on the lacrimal glands need further study.

The effects of air pollution on various clinical parameters of DED are different. Exposure to air pollution for 1 week had a greater effect on ocular discomforts and signs than exposure for 1 day or 1 month. However, the influences on the MG and tear cytokines were apparently in 1 month and 1 day, respectively. Different air pollutants also play diverse roles in different ocular characteristics. Exposure to high SO_2_ levels were more likely to cause ocular surface discomfort and damage as well as tear film instability, and the effects peaked within a week. While high NO_2_ levels were closely associated with MG functions and inflammatory cytokines and had a greater effect for 1 month. PM and O_3_ showed wide influences on the ocular surface. Li et al. and Tan et al. have found obvious dose–response relationships in the continuous exposure to air pollutants in animal models (17, 18). However, the concentrations of air pollution changes persistently from time to time. Inevitably, we have to use the mean concentrations in the present study. And our patients were asked to do 3–4 h outdoor activities in the corresponding zone. Thus, the dose–response relationship in this study seems not as evident as in those animal eyes. However, exposure to high levels of air pollution for 1 day can sufficiently upregulate inflammatory cytokines, 1-week exposure can obviously aggravate DED and 1-month exposure can apparently impair MG.

This study had several limitations. First, the study sample size was not large enough, which made it difficult to stratify the differences in DED subtypes for further analyses. Second, since this was a prospective cohort study, the results did not definitively provide causal evidence for the relationship between DED and air pollutants. Third, air-quality monitoring did not yield constant results, and there were differences between the indoor and outdoor activities of individuals. To avoid this discrepancy as much as possible, our participants were required to do 3–4 h outdoor activities in the corresponding zone. Fourth, the chemical characteristics of the compounds adsorbed to the particle surface will definite determine the PM toxic effects on the ocular surface and the correlation with DED symptoms, and those different effects will be clarified in further studies. Despite the above limitations, the present study is a well-designed multicenter prospective clinical study with organized statistical analysis. We have adjusted for several confounding factors including humidity and found consistent results both in the single and multi-pollutant models. We also considered the MGD and conducted laboratory examinations of inflammation in this study. Therefore, this present study still has some meaningful effects.

## Conclusion

In conclusion, increased PM_2.5_, O_3_, and SO_2_ exposures could cause ocular discomfort and damage as well as tear film instability. Increased PM_10_ concentration impaired tear film stability and ocular surface balance, however, it was not associated with eye symptoms. High O_3_ and NO_2_ concentrations decreased tear secretion, increased PM_2.5_ levels increased ST while reduced TMH. Exposure to high levels of air pollutants also impaired MG and upregulated tear cytokine concentrations. Thus, air pollutants seem to affect DED *via* various mechanisms. Furthermore, exposure to air pollutants for 1 week before the examination had the greatest effects on the symptoms and signs of DED, while exposure for 1 month and 1 day showed more obviously influences on MG and inflammatory cytokines, respectively. The time windows of air pollutants on different DED parameters were diversity. Further prospective multi-center clinical studies with large amounts of subjects from diverse regions are needed, such as severity classification, individual monitoring, personalized treatments, and longer follow-up periods.

## Data Availability Statement

The raw data supporting the conclusions of this article will be made available by the authors, without undue reservation.

## Ethics Statement

The studies involving human participants were reviewed and approved by Peking University Third Hospital Ethics Committee (No. M2019101). The patients/participants provided their written informed consent to participate in this study.

## Author Contributions

RH and MZ setup the protocol and recruited the participants. RH collected and analyzed the data, created the figures, and contributed to the writing. MZ and LZ discussed the data and participated in writing manuscript. YL, MS, JD, YX, FW, JW, XX, ZL, and SL recruited the participants. XL setup the protocol, and oversaw the final manuscript. All authors contributed to the article and approved the submitted version.

## Conflict of Interest

The authors declare that the research was conducted in the absence of any commercial or financial relationships that could be construed as a potential conflict of interest.

## Publisher’s Note

All claims expressed in this article are solely those of the authors and do not necessarily represent those of their affiliated organizations, or those of the publisher, the editors and the reviewers. Any product that may be evaluated in this article, or claim that may be made by its manufacturer, is not guaranteed or endorsed by the publisher.
